# Age-related changes in METS-IR and HOMA-IR in obese adults and their relationship with cardiometabolic comorbidities

**DOI:** 10.20945/2359-4292-2026-0059

**Published:** 2026-06-08

**Authors:** Esra Suay Timurkaan, Gülsüm Altuntaş, Muhammed Fuad Uslu, Hakan Ayyıldız, Mustafa Timurkaan

**Affiliations:** 1 Department of Internal Medicine Clinic, Fethi Sekin City Hospital, Elazig, Turkey; 2 Department of Intensive Care Clinic, Fırat University, Elazig, Turkey

**Keywords:** HOMA-IR, METS-IR, obesity, insulin resistance, comorbidity burden.

## Abstract

**Objective:**

Insulin resistance (IR) is central to cardiometabolic risk in obesity, but the clinical utility of IR indices may vary by age. We compared homeostasis model assessment for insulin resistance (HOMA-IR) and metabolic score for insulin resistance (METS-IR) across age groups in obese adults and examined comorbidity burden and discriminatory performance for type 2 diabetes mellitus (T2DM) and hypertension (HT).

**Subjects and methods:**

This retrospective, single-center study included adults with a BMI ≥ 30 kg/m^2^ (n = 481) across four age strata (18-40, 40-65, 65-75, and ≥75 years). Spearman correlation assessed the association between HOMA-IR and METS-IR. Comorbidity burden (0-3 chronic conditions) was modeled using ordinal logistic regression per 1-SD increase in z-scores, adjusted for age, sex, and BMI. Receiver operating characteristic analysis and multivariable logistic regression assessed T2DM and HT.

**Results:**

HOMA-IR and METS-IR were moderately correlated (ρ = 0.342 and *p* < 0.001, respectively). METS-IR was associated with comorbidity burden (adjusted odds ratio [OR] 1.84; 95% confidence interval [CI] 1.26-2.69; *p* < 0.01), whereas HOMA-IR was not (OR 1.12; 95% CI 0.92-1.36; *p* = 0.256). Discriminatory performance was limited for T2DM (area under curve [AUC] 0.500 vs. 0.537) and HT (AUC 0.471 vs. 0.519). METS-IR remained associated with T2DM (OR 2.51; 95% CI 1.60-3.93; *p* < 0.001) and HT (OR 1.81; 95% CI 1.13-2.91; *p* < 0.05).

**Conclusion:**

In obese adults, METS-IR demonstrated a stronger association with comorbidity burden than HOMA-IR. However, because both indices showed limited discriminatory performance for T2DM and HT, they should not be used as standalone tools for treatment guidance or diagnostic classification. Rather, they should be interpreted alongside age, clinical, and biochemical findings, as isolated use may be misleading and fail to identify disease in specific patient subgroups.

## INTRODUCTION

Obesity involves more than excess adipose tissue; it affects metabolic, hormonal, and inflammatory processes, predisposing individuals to insulin resistance (IR). Insulin resistance plays a central role in the pathogenesis of metabolic syndrome components such as type 2 diabetes mellitus (T2DM), dyslipidemia, hypertension (HT), and cardiovascular disease (^1^,^2^). The hyperinsulinemic-euglycemic clamp test, accepted as the gold standard for evaluating IR, is impractical for routine practice as it requires specialized equipment, trained personnel, and significant time (^3^). Consequently, indirect indices based on fasting glucose and insulin values, such as the homeostasis model assessment for insulin resistance (HOMA-IR), are preferred in clinical and epidemiological studies. Although HOMA-IR provides a practical measurement, its values vary by age, sex, body mass index (BMI), and population (^4^).

These limitations have driven the development of alternative indices that do not require insulin measurement. The metabolic score for insulin resistance (METS-IR) estimates IR by combining fasting glucose, triglycerides, HDL cholesterol, and BMI using a logarithmic formula (^5^). The fact that it does not require insulin levels, is based on commonly used biochemical parameters, and is less costly makes it an attractive option for routine practice. In addition, METS-IR is a strong predictor of metabolic syndrome and cardiovascular disease risk (^6^).

Age-related metabolic changes complicate the assessment of IR. Declining pancreatic β-cell reserve, loss of muscle mass, and altered adipose tissue distribution influence age-related insulin sensitivity. Based on this, it is plausible that HOMA-IR changes with age (^7^). Duan and cols. (^8^) analyzed NHANES 2001-2018 data and demonstrated a positive association between METS-IR and all-cause mortality. This association was particularly pronounced in individuals under 65 years of age and was stronger than that of HOMA-IR (^8^). This finding suggests that METS-IR varies by age and may have different predictive utility across age groups. In obese individuals, comparing HOMA-IR and METS-IR scores is important not only in terms of how accurately they reflect IR, but also in terms of how stable they are according to age and to what extent they provide benefit in predicting comorbidities. In the current literature, few large-scale studies have directly compared these indices across age groups in obese populations.

This study compared HOMA-IR and METS-IR in obese individuals across age strata to examine the effect of age on these scores. We also evaluated the performance of both indices in distinguishing accompanying diseases, primarily T2DM and HT. Thus, we aimed to determine the age-related stability, sensitivity, and clinical utility of these insulin-based and non-insulin-based formulas.

## SUBJECTS AND METHODS

### Study design and patient selection

This retrospective, observational, single-center study reviewed the records of adult patients who presented to the Internal Medicine outpatient clinics of Elazig Fethi Sekin City Hospital between January 1, 2020, and January 1, 2025. The inclusion criteria comprised patients with a BMI ≥30 kg/m^2^ and complete demographic, clinical, and laboratory data available in the hospital’s electronic information management system. Patients with missing, incomplete or unreliable data were excluded. In order to ensure the reliability of the results, we used strict criteria to minimize common confounding conditions and ensure that IR indices were evaluated within a more homogeneous obese population. Hence, we excluded patients if they were using insulin for diabetes, receiving immunosuppressive or chronic steroid therapy, had known chronic inflammatory disease or thyroid dysfunction, were in the early postoperative follow-up period, had advanced heart failure or advanced hepatic or renal dysfunction, had active psychiatric disease requiring treatment, had a history of malignancy, or were pregnant. After all data were obtained, the cohort was categorized into four age groups: Group 1 (18-40 years; young adult), Group 2 (40-65 years; middle-aged), Group 3 (65-75 years; older), and Group 4 (≥75 years; advanced age). This classification served as the primary grouping variable.

### Data collection and measurements

We extracted age, sex, height, body weight, fasting plasma glucose (mg/dL), triglycerides (mg/dL), LDL cholesterol (LDL-C, mg/dL), HDL cholesterol (HDL-C, mg/dL), total cholesterol (mg/dL), hemoglobin A1c (HbA1c, %), and fasting insulin (mIU/L) from the laboratory system. The BMI was calculated as weight (kg) divided by height (m^2^).

### Calculation of insulin resistance indices

Two indices were used to evaluate IR in obese individuals. The HOMA-IR was calculated using fasting insulin and fasting glucose levels using **Equation 1** (^9^):


(1) HOMA-IR=(fasting insulin [µU/mL] × fasting glucose [mg/dL])
/ 405

The METS-IR is a composite score derived from glucose, triglycerides, HDL-C, and BMI values; it was calculated using **Equation 2** (^5^):


(2) METS-IR=ln[2 × glucose (mg/dL) + triglycerides (mg/dL)] ×
BMI / ln[HDL-C (mg/dL)]

Both scores were analyzed as continuous variables and standardized into z-scores for modeling to ensure comparability.

### Variable definitions and comorbidity coding

T2DM and HT were defined as binary variables according to definitive ICD codes and active treatment records. In the raw table, the T2DM and HT variables were coded as 1 (diagnosis present) and 2 (diagnosis absent); in the analyses, these variables were recoded as 1 (present) and 0 (absent). Comorbidity burden was created by counting the number of predefined chronic diseases in the patient files. Comorbidity burden was calculated based on the presence of HT, T2DM, dyslipidemia, coronary artery disease, cerebrovascular disease, chronic kidney disease, chronic liver disease, and chronic lung disease. Each diagnosis was coded as 0 (absent) and 1 (present) and summed. In the analyses, this variable was used both for descriptive purposes and as an ordinal outcome variable. Sex was coded as a binary variable (1 = female, 2 = male). Age was analyzed as both a continuous variable and a categorical variable based on the four predefined strata.

### Statistical analyses

Statistical analyses were performed using R software (version 4.5.1; RStudio). Continuous variables were summarized as medians with interquartile ranges [IQR], and categorical variables as counts and percentages.

### Correlation analyses

Spearman’s rank correlation coefficient (ρ) assessed the relationship between HOMA-IR and METS-IR in the overall cohort and within each age group. Correlations between age and both indices were similarly evaluated.

### Ordinal logistic regression

Ordinal logistic regression models examined the association between comorbidity burden (range: 0-3) and the HOMA-IR and METS-IR scores. In these models, the number of comorbidities was the dependent variable, with HOMA-IR or METS-IR z-scores as the primary independent variables. Age (continuous), sex, and BMI were included in the models as covariates, and the results were reported as odds ratios (OR) with 95% confidence intervals (CI) per 1 standard deviation (SD) increase in the score.

### Receiver operating characteristic analyses

The receiver operating characteristic curves were generated to assess the performance of HOMA-IR and METS-IR for T2DM and HT. Area under the curve (AUC) values and 95% CIs were calculated using the pROC package and compared via the DeLong method. The positive class was defined as the presence of T2DM or HT in the relevant analysis.

### Logistic regression models

Multivariable logistic regression evaluated the independent effects of HOMA-IR and METS-IR z-scores for T2DM and HT, with the dependent variables being T2DM and HT, respectively, the main independent variable was the HOMA-IR z-score or METS-IR z-score, and the covariates were age (continuous), sex, and BMI. Results were presented as ORs, 95% CIs, and *p*-values.

### Linear regression analyses

To evaluate the linear relationship between age and IR scores in more detail, multivariable linear regression models were constructed with HOMA-IR and METS-IR separately as dependent variables. In these models, age was the main independent variable, and sex and BMI were included as covariates. In addition, age×HOMA-IR and age×METS-IR interaction terms were added to supplementary models for T2DM to test whether the associations of the scores with T2DM changed significantly by age.

### Missing data management

Because records with missing data were excluded during patient selection, a complete-case approach was used for all models.

### Visualization

The ggplot2 package generated scatter plots, box plots by age group, and distribution plots by comorbidity burden. Trend lines for continuous variables were fitted using locally estimated scatterplot smoothing with 95% CIs.

### Statistical significance

All tests were two-sided, with *p* < 0.05 considered statistically significant. No adjustments were made for multiple comparisons; thus, findings are considered exploratory.

## RESULTS

### Clinical and demographic characteristics

Participants were distributed relatively evenly across the four age groups (**Table 1**). Females predominated in all age groups. Median BMI ranged from 34.6 to 35.6 kg/m^2^ and showed no clear trend with advancing age. Glucose and triglyceride levels were higher in the 40-65 and 65-75 age groups, although these differences remained within expected limits for the participants’ BMI and metabolic status. In the overall cohort, T2DM prevalence was 25.8% (n = 124). By age group, T2DM prevalence was 5.3% (7/132) in the 18-40 group, 32.5% (41/126) in the 40-65 group, 33.1% (41/124) in the 65-75 group, and 35.4% (35/99) in the ≥75 group. Similarly, HT was present in 16.2% (n = 78) of the cohort. Across age groups, HT prevalence was 3.0% (4/132) in the 18-40 group, 23.0% (29/126) in the 40-65 group, 18.5% (23/124) in the 65-75 group, and 22.2% (22/99) in the ≥75 group (**Table 1**).

**Table 1:** Clinical and demographic characteristics of the obese cohort by age group

Variable	Age group (years)			
	18-40	40-65	65-75	>75
Individuals (n)	132	126	124	99
Female, n (%)	107 (81.1)	107 (84.9)	103 (83.1)	77 (77.8)
Diabetes mellitus, n (%)	7 (5.3)	41 (32.5)	41 (33.1)	35 (35.4)
Hypertension, n (%)	4 (3.0)	29 (23.0)	23 (18.5)	22 (22.2)
≥2 comorbidities, n (%)	4 (3.0)	27 (21.4)	22 (17.7)	14 (14.1)
Age (years)	30.0 (22.0-37.0)	51.0 (45.0-58.0)	68.0 (66.0-70.0)	78.0 (75.0-86.0)
BMI (kg/m^2^)	35.5 (32.0-40.2)	35.6 (32.5-41.0)	34.7 (32.0-38.1)	34.6 (31.3-39.9)
Glucose (mg/dL)	87.5 (82.0-95.0)	96.5 (86.2-126.8)	99.0 (88.0-117.0)	103.0 (87.0-116.0)
Triglycerides (mg/dL)	113.5 (85.5-170.5)	134.5 (95.5-200.0)	134.0 (93.8-164.0)	118.0 (96.5-159.5)
LDL-C (mg/dL)	105.0 (90.0-126.0)	112.5 (95.0-135.2)	118.5 (100.0-145.0)	108.0 (91.5-138.5)
HDL-C (mg/dL)	44.5 (36.0-51.0)	48.0 (43.0-55.0)	47.0 (40.0-57.2)	44.0 (39.0-52.0)
Total cholesterol (mg/dL)	174.5 (154.2-201.0)	196.0 (171.2-228.8)	195.0 (169.0-226.5)	186.0 (159.5-220.0)
HbA1c (%)	5.5 (5.3-5.8)	6.0 (5.6-6.6)	6.0 (5.7-6.7)	6.1 (5.6-6.4)
Insulin (mIU/L)	15.0 (10.1-23.4)	11.9 (9.2-18.3)	10.4 (7.3-16.2)	10.2 (6.0-16.0)
HOMA-IR	3.4 (2.3-5.3)	3.3 (2.1-5.2)	2.7 (1.8-4.3)	2.6 (1.4-4.3)
METS-IR	44.2 (38.9-49.9)	45.2 (39.7-51.0)	42.5 (39.3-47.6)	43.1 (40.1-48.8)

### HOMA-IR and METS-IR levels and their relationship

**Figure 1:**
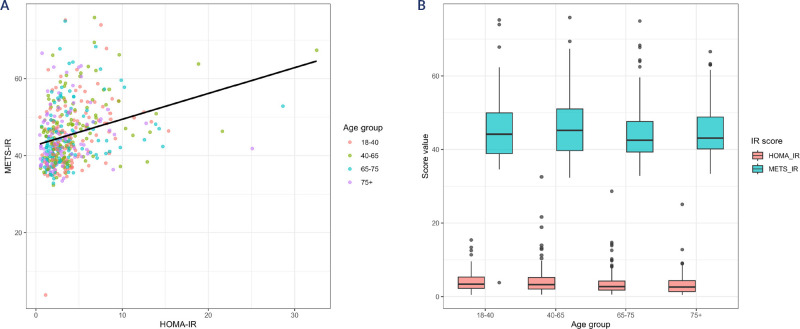
**A)** Scatter plot of METS-IR versus HOMA-IR by age group; each point represents one participant. **B)** Distributions of HOMA-IR and METS-IR across age groups; box plots depict the distributions of HOMA-IR (above) and METS-IR (below) by age group. Boxes represent the interquartile range, horizontal lines denote the median, and whiskers represent the approximate data range.

A moderate positive correlation between HOMA-IR and METS-IR emerged in the overall cohort (Spearman *p* = 0.34; *p* < 0.001). In age-stratified analyses, this correlation remained significant across all groups (*p* = 0.32, 0.37, 0.39, and 0.29 for the 18-40, 40-65, 65-75, and ≥75 age groups, respectively; all *p* < 0.01) (**Table 2**). A scatter plot demonstrated an upward trend between HOMA-IR and METS-IR across the obese cohort (**Figure 1A**). Box plots by age group revealed no marked shift in HOMA-IR or METS-IR distributions from young adulthood to advanced age, although the distributions became more heterogeneous in older groups (**Figure 1B**).

**Table 2:** Spearman correlations between HOMA-IR and METS-IR overall and by age group

Age group (years)	Spearman rho	*p*-value
Overall	0.342	<0.001
18-40	0.321	<0.001
40-65	0.366	<0.001
65-75	0.393	<0.001
>75	0.289	<0.01

Spearman correlation coefficients (ρ) and *p*-values are shown for the overall cohort and each age stratum.

### Association between HOMA-IR and METS-IR and comorbidity burden

METS-IR levels increased more consistently as the number of comorbidities increased, whereas no clear stepwise pattern was observed for HOMA-IR (**Table 3A**). In individuals without comorbidities, the median HOMA-IR was 3.0 (IQR: 2.01-4.55), while it was 2.52 (IQR: 1.57-5.02) in those with three comorbidities. Median METS-IR was 43.6 (IQR: 39.3-49.3) in participants without comorbidities and 44.5 (IQR: 41.2-50.7) in those with three comorbidities (**Table 3A**). Although absolute differences were limited, METS-IR showed a distinct and consistent upward trend as the number of comorbidities increased.

**Table 3A:** Distribution of HOMA-IR and METS-IR across comorbidity categories

comorb_ord	n	HOMA-IR, median (IQR)	METS-IR, median (IQR)
0	335	3.0 (2.01-4.55)	43.6 (39.3-49.3)
1	79	2.96 (1.46-5.61)	45.0 (38.9-51.6)
2	52	3.13 (2.11-4.49)	43.1 (40.7-47.7)
3	15	2.52 (1.57-5.02)	44.5 (41.2-50.7)

Comorbidity categories are defined as the total number of documented chronic conditions (0-^3^). Values are presented as n and median (interquartile range).

**Table 3B:** Ordinal logistic regression for comorbidity burden

Predictor	OR	95% CI	*p*-value
HOMA-IR z-score	1.12	0.92-1.36	0.256
METS-IR z-score	1.84	1.26-2.69	<0.01

Ordinal logistic regression models estimate the odds of being in a higher comorbidity category per 1 standard deviation increase in HOMA-IR or METS-IR z-scores, adjusting for age, sex, and BMI.

**Figure 2:**
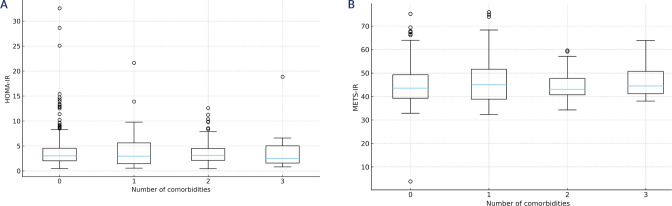
**A)** HOMA-IR by number of comorbidities; box plots showing HOMA-IR distributions across increasing comorbidity counts. A modest increase is observed with greater comorbidity burden, although overlapping between categories remained substantial. **B)** METS-IR by number of comorbidities; box plots showing the distributions of METS-IR across comorbidity categories. Unlike HOMA-IR, METS-IR showed a more monotonic trend across comorbidity categories, consistent with its association with overall cardiometabolic burden.

In ordinal logistic regression, a 1-SD increase in the HOMA-IR z-score did not significantly increase the likelihood of transitioning to a higher comorbidity category (OR = 1.12; 95% CI: 0.92-1.36; *p* = 0.256). Conversely, a 1-SD increase in the METS-IR z-score increased this likelihood approximately 1.8-fold (OR = 1.84; 95% CI: 1.26-2.69; *p* < 0.01). Box plots visually supported the more pronounced upward shift in METS-IR with increasing comorbidities (**Figures 2A** and **2B**).

### Diagnostic performance of HOMA-IR and METS-IR for T2DM and HT

**Figure 3:**
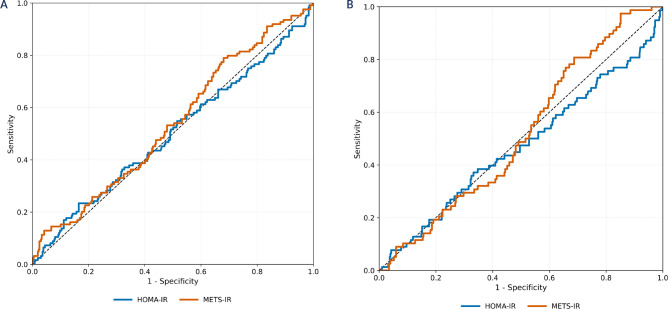
**A)** ROC curves of HOMA-IR and METS-IR for diabetes; ROC curves for diabetes, wherein HOMA-IR AUC: 0.500 (95% CI 0.439-0.562) and METS-IR AUC: 0.537 (95% CI 0.478-0.595). The diagonal line indicates no discrimination. **B)** ROC curves of HOMA-IR and METS-IR for hypertension. ROC curves for hypertension, wherein HOMA-IR AUC: 0.471 (95% CI: 0.396-0.546); METS-IR AUC: 0.519 (95% CI: 0.455-0.583). The diagonal line indicates no discrimination.

In the obese cohort, HOMA-IR demonstrated limited discriminative ability for T2DM (AUC 0.500; 95% CI 0.439-0.562), and logistic regression revealed no significant association between a 1-SD increase and the odds of T2DM (OR 1.12; 95% CI 0.91-1.37; *p* = 0.290) (**Table 4, Figure 3A**). The AUC value of METS-IR was 0.537 (95% CI 0.478-0.595), and its discriminative performance was also limited. However, each 1-SD increase was significantly associated with the odds of T2DM (OR 2.51; 95% CI 1.60-3.93; *p* < 0.001) (**Table 4, Figure 3A**). For HT, the AUC of HOMA-IR was 0.471 (95% CI 0.396-0.546) and its OR was not significant (OR 1.03; 95% CI 0.80-1.31; *p =* 0.814); the AUC of METS-IR was 0.519 (95% CI 0.455-0.583), and while its discriminative ability remained limited, each 1-SD increase was associated with the odds of HT (OR 1.81; 95% CI 1.13-2.91; *p* < 0.05) (**Table 4, Figure 3B**). Overall, the AUC values near 0.5 indicated that both indices had limited discriminative capacity for T2DM and HT.

**Table 4:** Discriminative performance of HOMA-IR and METS-IR for T2DM and HT

Outcome	Model	AUC	AUC 95% CI	OR per 1 SD	OR 95% CI	*p*-value
T2DM	HOMA-IR	0.500	0.439-0.562	1.12	0.91-1.37	0.290
	METS-IR	0.537	0.478-0.595	2.51	1.60-3.93	<0.001
HT	HOMA-IR	0.471	0.396-0.546	1.03	0.80-1.31	0.814
	METS-IR	0.519	0.455-0.583	1.81	1.13-2.91	<0.05

The area under curve (AUC) values and 95% confidence intervals (CI) are derived from ROC analyses using HOMA-IR and METS-IR as continuous predictors. Odds ratios (OR) are estimated per 1 standard deviation increase in the corresponding z-scores, adjusted for age, sex, and BMI.

### Change in HOMA-IR and METS-IR with age

**Figure 4:**
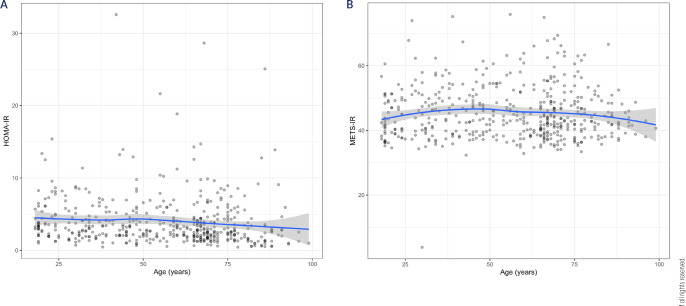
**A)** Relationship between age and HOMA-IR. Scatter plot showing individual HOMA-IR values across age groups. The LOESS smoothing curve is overlaid. **B)** Relationship between age and METS-IR; Scatter plot showing METS-IR values by age. The LOESS smoothing curve is overlaid.

A weak negative correlation between age and HOMA-IR emerged in the overall obese cohort (Spearman *p* = -0.196; *p* < 0.001; **Table 5**). In multivariable linear regression adjusted for age, sex, and BMI, each 1-year increase in age was associated with a mean 0.016-unit decrease in HOMA-IR (95% CI -0.030 to -0.001; *p* = 0.045) (**Table 5**). Curve plots of the age distribution supported this trend for HOMA-IR (**Figures 4A** and **4B**). The correlation between METS-IR and age was not statistically significant (Spearman *p* = -0.020; *p* = 0.659) (**Table 5**). In the adjusted linear regression model, the effect of age on METS-IR was also non-significant (95% CI -0.0102 to 0.0199; *p* = 0.526) (**Table 5**). In models including an age × T2DM interaction, these associations did not differ by T2DM status (*p* = 0.149 for HOMA-IR; *p* = 0.745 for METS-IR) (**Table 5**). Curve plots indicated no significant age-related trend for METS-IR (**Figures 4A** and **4B**).

**Table 5:** Associations between age and insulin resistance indices

Score	Spearman rho	Spearman p	β_age (per year)	β 95% CI	*p* (linear regression)	Interaction *p* (age × DM status)
HOMA-IR	-0.196	<0.001	-0.016	-0.030--0.001	0.045	0.149
METS-IR	-0.020	0.659	0.0049	-0.010-0.019	0.526	0.745

Spearman correlations describe the monotonic relationship between age and each index. Linear regression estimates (β) reflect the average change in the index per 1-year increase in age, adjusted for sex and BMI. The interaction term tests whether the relationship between age and each index differs by diabetes status.

## DISCUSSION

The main finding of our study is that although METS-IR reflected comorbidity burden more strongly than HOMA-IR in obese adults, neither index demonstrated strong diagnostic utility for specific conditions in age-adjusted analyses. In addition, both indices provided only limited discriminative ability for T2DM and HT. Clinically, METS-IR serves as a non-insulin-based index reflecting cardiometabolic burden across the obesity spectrum.

Obesity induces IR through metabolic and hormonal alterations, and this resistance is central to the pathogenesis of metabolic syndrome (^10^). HOMA-IR and METS-IR are important indicators for evaluating IR, with METS-IR offering practical application and predictive power for cardiometabolic risk (^11^). Although BMI was relatively homogeneous across age groups, our findings showed that the prevalence of T2DM and HT increased markedly at older ages, which is consistent with large cohort studies suggesting that metabolically healthy obesity is rarely sustainable long-term (^12^-^15^). Our data support the observation that a significant proportion of obese individuals progress toward multiple cardiometabolic risk factors and organ damage, as evidenced by the age-related increase in clinical comorbidities.

Although HOMA-IR is frequently used to evaluate IR, its sensitivity can be influenced by age and sex. METS-IR has emerged as a promising alternative that may better reflect IR and predict cardiovascular disease risk in obese populations (^6^). In our population, although a statistically significant correlation was found between HOMA-IR and METS-IR in all age groups, this significance was not at levels that would be interpreted as a strong association. Nevertheless, the correlation coefficients not being high enough to completely overlap suggests that these two indices capture partially different dimensions of IR. Indeed, studies in different populations have shown that while METS-IR correlates moderately to highly with HOMA-IR, it often outperforms HOMA-IR in predicting long-term IR-related outcomes (^16^-^18^).

Hence, our study contributes by showing that, in a large cohort consisting of obese individuals and stratified by the age factor, this relationship remained relatively stable by age, albeit that the slope became clearly flatter in the most advanced age group. This suggests that while the HOMA-IR signal may weaken with age due to altered insulin secretion dynamics and body composition, METS-IR may provide more consistent information regarding metabolic stress via glucose and triglyceride levels. The concurrent rise in HOMA-IR and METS-IR with increasing comorbidities reflects the expected characteristics of increasing cardiometabolic burden together with IR in obese individuals. However, a 1-SD increase in the HOMA-IR z-score did not significantly increase the probability of a higher comorbidity count. In contrast, a 1-SD increase in the METS-IR z-score increased the likelihood of a higher comorbidity burden approximately 1.8-fold. The existing difference suggests that METS-IR behaves as a stronger risk marker in practice.

Recent studies have shown that METS-IR is associated with components of metabolic syndrome and target organ damage and, in some studies, has even been associated with subclinical atherosclerosis and clinical cardiovascular events (^16^,^19^-^21^). In fact, some researchers have concluded that the HOMA-IR score is a predictive factor for middle-aged and older populations (^22^). Others have reported that METS-IR predicts the development of HT, T2DM, and cardiovascular disease in populations with heterogeneous BMI distributions (^16^,^23^,^24^). Most of these studies were conducted in the general population or across mixed BMI groups. Our findings corroborate this literature within a cohort consisting only of obese individuals and using an easily measurable endpoint (e.g., number of comorbidities). The closer association between METS-IR and multiple comorbidities suggests that this index could be used in clinical practice as a dynamic risk score reflecting cumulative cardiometabolic burden and multimorbidity in obese patients.

The fact that the AUC of HOMA-IR and METS-IR for T2DM or HT were very close to 0.5 indicates that neither index is sufficient as a threshold-based diagnostic tool in obese individuals. These modest AUCs do not contradict the multivariable associations observed in our models, as association and discrimination quantify different statistical properties. In an obesity-only cohort, the restricted case-mix leads to overlapping score distributions between participants with and without T2DM or HT, limiting separability. Baseline cardiometabolic risk is uniformly high across the cohort, and a ceiling effect may further reduce discrimination. In addition, because outcomes were defined by cross-sectional diagnostic status, prior detection and treatment may have blurred intergroup differences. In contrast, some researchers who considered these outcomes to be clinically meaningful discrimination typically evaluated incident T2DM, HT, or cardiovascular events over long-term follow-up (^9^,^18^). Consequently, our results indicate that HOMA-IR and METS-IR cannot independently establish diagnostic thresholds for T2DM or HT in obese individuals. Instead, METS-IR is more appropriately interpreted as a chronic risk score reflecting the accumulation of comorbidities and cardiometabolic burden in obesity.

A study series consisting of adolescent obese individuals showed that the correlation of METS-IR with metabolic syndrome components and its strength in identifying IR persist across different ethnic and age groups (^25^-^27^). These findings suggest that METS-IR provides a signal of energy surplus that includes not only glycemic control but also impairments along the triglyceride and HDL cholesterol axes. In our study, the strong association between comorbidity count and METS-IR indicates that this risk signal translates into clinical disease burden in obese adults. Nonetheless, because our endpoint was the number of comorbidities and specific organ outcomes (e.g., chronic kidney disease or incident cardiovascular events) were not evaluated separately. Therefore, rather than directly comparing our diagnostic thresholds with previous studies, we emphasize that our findings provide a complementary perspective on cumulative risk.

Our study contributes to this discussion in two respects in that METS-IR shows a strong association with comorbidity burden in obese adults despite providing limited diagnostic performance for T2DM and HT. First, it demonstrates that even the best-performing indices may rapidly lose diagnostic discriminative ability in samples characterized by high baseline risk and phenotypic homogeneity. Second, it suggests that interpreting METS-IR within the family of non-insulin-based indices as a “general cardiometabolic burden score” may be a more realistic positioning than establishing a diagnosis of a single disease. Similarly, research in obese pediatric populations has shown that puberty-specific physiological IR necessitates age-specific HOMA-IR cut-off values (^28^). Although this study pertains to a pediatric population, the idea of the need for age-specific IR thresholds provides a relevant conceptual framework for evaluating HOMA-IR and METS-IR in obese adults. Our findings suggest a similar phenomenon emerges in older obese adults. We found that HOMA-IR decreased slightly with age, whereas METS-IR remained more stable and maintained its association with comorbidity burden. This suggests that using a single HOMA-IR or METS-IR cut-off value across all adult age groups is not clinically realistic in obese populations. Indeed, the literature indicates that age-adjusted thresholds or age-standardized scores yield more consistent results in this context (^22^,^29^,^30^).

Our findings may be interpreted as observational evidence supporting future studies aimed at determining age-specific IR thresholds, particularly given the context of sarcopenic obesity and changing body composition that may occur at older ages. Several key messages stand out regarding the clinical translation of our findings. First, even when HOMA-IR and METS-IR are elevated in obese individuals, using these scores in isolation to screen for T2DM or HT via fixed thresholds is not supported by our data. Second, the association between METS-IR and comorbidity burden suggests that integrating this score with BMI and routine biochemical parameters as a continuous risk indicator is clinically useful. Third, as age advances, caution is required particularly in interpreting HOMA-IR, and age-sensitive thresholds and long-term follow-up must be considered.

Despite this study’s findings, several limitations should be addressed. First, the single-center, retrospective, cross-sectional design limits causal inference and increases the likelihood that unmeasured clinical factors may have influenced the observed associations. Second, because the cohort consisted exclusively of obese adults evaluated in an outpatient clinic, the findings should be generalized cautiously and should not be directly extrapolated to non-obese populations, community-based screening cohorts, insulin-treated patients, or populations with substantially different demographic and metabolic profiles. Third, comorbidity burden was assessed using a count-based composite measure rather than organ-specific clinical outcomes, providing a practical but relatively coarse representation of disease burden. Fourth, the diagnostic analyses relied on cross-sectional disease status; prior diagnoses, treatments, and overlapping score distributions likely attenuated discrimination. Therefore, although our data support METS-IR as an adjunctive indicator of cardiometabolic burden in obese adults, they do not support its standalone use for threshold-based diagnosis. The external validity of these findings requires confirmation in prospective, multicenter cohorts with broader case-mix diversity. Despite these limitations, the study strengths include the concurrent evaluation of HOMA-IR and METS-IR within a single obese cohort spanning a broad adult age range, and the assessment of comorbidity burden using ordinal models that explicitly consider age interactions.

In conclusion, METS-IR demonstrated a stronger association with comorbidity burden than HOMA-IR in obese adults and remained more stable across age groups. METS-IR is a non-insulin-based continuous index derived from routinely available anthropometric and fasting laboratory measures. However, both METS-IR and HOMA-IR provided only limited discriminatory ability for T2DM and HT. Therefore, these indices should not be used as standalone diagnostic tools or isolated guides for treatment decision-making. Rather, their clinical role is more appropriate as adjunctive, continuous indicators of cardiometabolic burden when interpreted alongside age, phenotype, and other clinical and biochemical variables. Relying on these scores in isolation may be misleading in certain patient groups, as overlapping score distributions can reduce sensitivity and contribute to the under-recognition of clinically relevant disease. Given the single-center, retrospective, cross-sectional design and the restriction to obese outpatients, the broader applicability of these findings must be established in prospective, multicenter cohorts encompassing more diverse age groups and clinical profiles.

## Data Availability

datasets related to this article will be avail-able upon request to the corresponding author.
